# Prediction of Phase Behavior of Spray-Dried Amorphous Solid Dispersions: Assessment of Thermodynamic Models, Standard Screening Methods and a Novel Atomization Screening Device with Regard to Prediction Accuracy

**DOI:** 10.3390/pharmaceutics10010029

**Published:** 2018-03-07

**Authors:** Aymeric Ousset, Pierre-François Chavez, Joke Meeus, Florent Robin, Martin Alexander Schubert, Pascal Somville, Kalliopi Dodou

**Affiliations:** 1 School of Pharmacy and Pharmaceutical Sciences, Faculty of Health Sciences and Wellbeing, University of Sunderland, Sunderland SR13SD, UK; aymeric.ousset@gmail.com; 2 UCB Pharma S.A., Drug Delivery Design and Development, B-1420 Braine l’Alleud, Belgium; Pierre-Francois.Chavez@ucb.com (P.-F.C.); joke.meeus.1@gmail.com (J.M.); Florent.Robin@ucb.com (F.R.); Martin-Alexander.Schubert@gmx.net (M.A.S.); Pascal.Somville@ucb.com (P.S.)

**Keywords:** amorphous solid dispersions, miscibility, solid state properties, spray dryer, screening, polymers, Flory–Huggins theory, phase diagram

## Abstract

The evaluation of drug–polymer miscibility in the early phase of drug development is essential to ensure successful amorphous solid dispersion (ASD) manufacturing. This work investigates the comparison of thermodynamic models, conventional experimental screening methods (solvent casting, quench cooling), and a novel atomization screening device based on their ability to predict drug–polymer miscibility, solid state properties (*T*_g_ value and width), and adequate polymer selection during the development of spray-dried amorphous solid dispersions (SDASDs). Binary ASDs of four drugs and seven polymers were produced at 20:80, 40:60, 60:40, and 80:20 (*w*/*w*). Samples were systematically analyzed using modulated differential scanning calorimetry (mDSC) and X-ray powder diffraction (XRPD). Principal component analysis (PCA) was used to qualitatively assess the predictability of screening methods with regards to SDASD development. Poor correlation was found between theoretical models and experimentally-obtained results. Additionally, the limited ability of usual screening methods to predict the miscibility of SDASDs did not guarantee the appropriate selection of lead excipient for the manufacturing of robust SDASDs. Contrary to standard approaches, our novel screening device allowed the selection of optimal polymer and drug loading and established insight into the final properties and performance of SDASDs at an early stage, therefore enabling the optimization of the scaled-up late-stage development.

## 1. Introduction

New chemical entities with unfavorable water solubility properties are continuously emerging from high-throughput screening during the drug discovery phase in the pharmaceutical industry [1]. There are many problems resulting from the poor aqueous solubility of current pipeline candidates in drug development, such as limitations in formulation strategies and oral bioavailability during drug administration. According to the Biopharmaceutics Classification System (BCS), drugs are classified into four categories based on their solubility and intestinal permeability properties [2]. In recent decades, amorphous solid dispersions (ASDs) have become a common formulation strategy to increase the bioavailability of poorly water soluble compounds (class II&IV) [3].

ASDs were initially defined by Chiou and Riegelman (1971) as “a dispersion of one or more active ingredients in an inert carrier at the solid state, prepared by the melting, the solvent, or the melting solvent method” [4]. The role of the polymer is to stabilize the amorphous form of the drug by reducing its molecular mobility and hence preventing it from crystallization. The improvement of dissolution rate is mainly correlated to the increase of specific surface area and the decrease of diffusion layer thickness of the formulation. These characteristics are respectively induced by the generated porosity, drug particle size reduction, and improved wettability [5]. The apparent solubility is also enhanced with the presence of a potential amorphous state within the drug. Numerous relevant publications outlined the improved solubility and dissolution rate of ASDs in comparison to crystalline drug suspensions [6]. However, the inherent physicochemical instability of ASDs—in particular their tendency to crystallization—restrains the number of marketed amorphous products (Sporanox^®^ (Itraconazole) in 1992, Intelence^®^ (Etravirine) in 2008, Noxafil^®^ (Posaconazole) in 2013). Among the solvent-based technologies, spray drying is a suitable and scalable process for ASD production from laboratory to commercial scale [7].

Drug–polymer miscibility is a critical parameter that affects the performance (e.g., physical stability upon storage and dissolution rate) of the formulated product. The term *miscibility* refers to the presence of a single amorphous drug–polymer phase [8]. The combination of crystalline drug with amorphous polymer can generate multiple phase behaviors after processing; this can result in the presence of crystalline or amorphous drug suspensions in the carrier and a glass solution [9]. The latter is the most desirable state, where the drug is molecularly dispersed into the carrier and ensures bioavailability enhancement. Therefore, the inherent solid state of spray-dried amorphous solid dispersions (SDASDs) is a complex combination of the nature of the carrier, solvent choice, drug crystallization tendency, drug loading (DL), drug–polymer interactions, and process manufacturing conditions [10].

The selection of an optimal drug–polymer combination and DL in the early stage of drug formulation would ensure successful formulation development. This impacts several properties of the final formulation, such as glass transition temperature (*T*_g_), phase behavior, particle morphology, wettability, dissolution performance, hygroscopicity, molecular mobility, and hence solid state stability [10]. The possibility of interactions between drug and carrier is an additional advantage, as specific interactions might increase the solubility of the drug in the carrier and consequently impede phase separation and nucleation/crystallization [11]. 

The prediction of drug–polymer miscibility using theoretical considerations has been widely reported in the literature, including the estimation of solubility parameter [12], Flory–Huggins (FH) coefficient [13], Greenhalg criterion [14], Bagley plot [15], melting point (*T*_m_) depression [16], phase diagram [17], computational tools [18], and perturbed-chain statistical associating fluid theory (PC-SAFT) [19]. In addition to these thermodynamic models, Duarte et al. (2015) refined a new computational tool by implementing kinetics and manufacturing considerations to the initial theory of mixing [20]. Despite their use, limitations and inaccuracies of thermodynamic models have already been demonstrated [21]. In the case of spray drying, fast solvent evaporation would favor the formation of a kinetically frozen amorphous system which generally exceeds crystalline drug solubility and amorphous drug miscibility in the polymer [22].

Many experimental screening methodologies have been developed with regards to development constraints; i.e., the limited amount of compound, time constraints, and the ability to predict how formulation attributes will evolve during the scaling-up phase in pharmaceutical development [23]. Continuous efforts for miniaturization, high-throughput automation, and Quality by Design approach have increased the number of excipients tested and allowed a larger number of tests to be performed in parallel [24]. Among the different works, screening strategies investigated both the solid state, the stability under stress conditions, and also the solubility-enhancing capabilities of ASDs [25]. Recent studies have been oriented towards improving the understanding of small-scale drying processes via the use of electrospraying or levitated droplet drying kinetics [26,27]. 

Unfortunately, polymer selection, DL, and manufacturing process are still iteratively and empirically driven. Theoretical models have been used in combination with various screening methodologies for the purpose of carrier selection in early drug development phases. However, no systematic approach to evaluating the prediction accuracy of theoretical and experimental screening methodologies in terms of drug–polymer miscibility prediction, and therefore appropriate polymer (and DL) selection for SDASD manufacturing, has been conducted so far. Additionally, due to its capacity to operate in a fully-automated mode, solvent casting is the most frequently used screening procedure in the early stages of drug development. However, previous studies have identified that the preparation method during solvent casting affects the phase behavior of solid dispersions and hence influences their drug release kinetics [28] and physical stability upon storage. Specifically, the difficulty of removing the last fraction of residual solvent from film-casted samples remains one of the main constraints of this approach, since it reduces the mixing *T*_g_ of the system and the kinetic glass stability. In this regard, there is a particular interest in implementing an alternative screening approach able to provide a reliable insight into the final properties of SDASDs so that a stronger correlation between the preformulation bench screening and the batch-scale production can be established.

The present work aims to provide guidance for the accurate prediction of the phase behavior of spray-dried samples that would lead to adequate polymer selection. This aim is achieved via the systematic investigation and comparison of the prediction accuracy of theoretical thermodynamic models (theory of mixing), experimental screening methods (e.g., film casting and quench cooling), and of a novel atomization screening device. This study focused on four model drugs and seven polymers to gain a better understanding of both critical formulation attributes and critical process parameters for designing robust SDASDs. The selection of a large set of polymers with different chemical and physical properties was done in compliance with the typical list of carriers tested during preformulation screening in the pharmaceutical industry. The solid state and phase behavior of formulations were characterized systematically by modulated differential scanning calorimetry (mDSC) and X-ray powder diffraction (XRPD). The influence of solvent evaporation rate was investigated by comparing the miscibility of film-casted ASDs performed at various evaporation speeds to spray-dried formulations. The different screening methods were compared based on their ability to predict: (i) drug–polymer miscibility; (ii) solid state properties of spray-dried formulations (T_g_ value and width); and (iii) polymer rank order for SDASD development. Principal component analysis (PCA) was used to qualitatively compare and rank the screening approaches based on the three criteria cited above, which to our knowledge has not been done to-date.

## 2. Materials and Methods

### 2.1. Material

#### 2.1.1. Model Drugs

**Figure 1:**
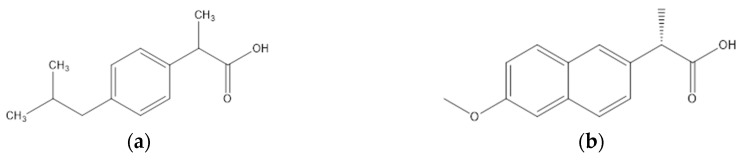

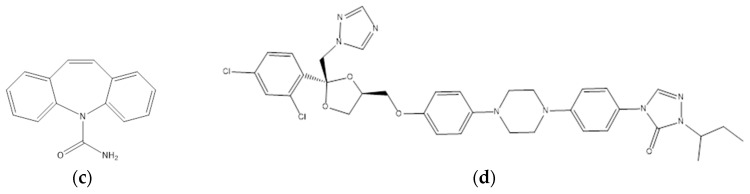
Chemical structure of (**a**) Ibuprofen; (**b**) Naproxen; (**c**) Carbamazepine; and (**d**) Itraconazole.

The chemical structures of the model drugs used in this study are illustrated in Figure 1. 

The choice of four BCS class II model drugs was based on their similar/different properties in term of chemical structure, H-bond donor/acceptor sites, molecular weight, crystallization tendency, and thermal properties as reported in Table 1. The chemical properties were determined using MarvinSketch software (ChemAxon, Budapest, Hungary, version 6.3.0). The thermal properties were experimentally characterized using mDSC and thermogravimetric analysis TGA/SDTA 851e (Mettler Toledo, Columbus, OH, USA). Glass forming ability (GFA)/glass stability (GS) class was verified from the available literature [29]. 

Crystalline ibuprofen (IBU) was donated by BASF (Ludwigshafen am Rhein, Germany). Crystalline naproxen (NAP), carbamazepine (CAR), and itraconazole (ITR) were purchased from SRIS Pharmaceuticals (Hyderabad, India).

#### 2.1.2. Polymers

The physico-chemical properties of the selected polymers are reported in Table 2. The chemical properties were obtained from the Handbook of Pharmaceutical Excipients, Fifth Edition (2006) [30]. Thermal properties were experimentally characterized using mDSC and TGA. 

Hydroxypropylmethylcellulose phthalate (HPMCP-HP50, henceforth HP50) and hydroxypropylmethylcellulose acetate succinate fine grade (HPMCAS-LF, hence HAS) were obtained from Shin-Etsu (Tokyo, Japan). PVPVA (the copolymer of *N*-vinyl-2-pyrrolidone and vinyl acetate, henceforth PVA) was obtained from Ashland (Covington, KY, USA), and polyvinylpyrrolidone (PVPK30, hence PK30) was obtained from VWR (Radnor, PA USA). Soluplus (SOL, the copolymer of polyvinyl caprolactam-polyvinyl acetate-polyethylene glycol graft co-polymer) was donated by BASF (Ludwigshafen am Rhein, Germany). Eudragit L100 (EUD, 1:1 copolymer of methacrylic acid, methyl methacrylate) and Eudragit L100-55 (EUD55, 1:1 copolymer of methacrylic acid, ethyl acrylate copolymer) were donated by Evonik (Essen, Germany). The solvents used were of analytical or HPLC grade.

### 2.2. Methods

#### 2.2.1. Theoretical Models Based on the Thermodynamics of Mixing

Initially developed for polymer–solvent mixtures, FH theory has frequently been adapted to ASD systems. The main interest of such a model is to take into account the non-ideal entropy of mixing resulting from the combination of a large and a small molecule [31]. The Gibbs free energy of mixing may be expressed as a function of FH coefficient, as detailed in Equation (1):

(1) $$\frac{\Delta G_{mix}}{RT}=\emptyset_{Drug}\times \ln\left(\emptyset_{Drug}\right)+\frac{\emptyset_{Pol}}{m}\times \ln\left(\emptyset_{Pol}\right)+\chi_{Drug-Pol}\times \emptyset_{Drug}\times \emptyset_{Pol},$$

where Δ*G_mix_* is the free energy of mixing, *Φ* is the volume fraction, *χ* is the FH coefficient, and *m* is the ratio of the volume of a polymer chain to the volume of the lattice. The molar volume of the drug is commonly used as the volume of the lattice site [12].

Favorable mixing conditions between the two entities are induced by negative values of Gibbs free energy. Under thermodynamically favorable mixing conditions, the solubility of the system composition is lower than the equilibrium solubility, and the resulting solid dispersion will form a homogenous solution. The enthalpic contribution is represented by the last term on the right-hand side of Equation (1). The combination of a small molecule with a polymer results in an entropic term favorable to the mixing. Thus, the value of the enthalpic term will dictate the sign of the Gibbs free energy. A negative or slightly positive value of FH parameter will lead to a favorable enthalpy of mixing.

The stability of the system can be assessed by evaluating the sign of the first- and second-order derivatives of the free energy of mixing. The highest drug–polymer miscibility is determined using the spinodal curve (second derivative) as shown in Equation (2), and represents the boundary point between metastable and unstable region at a given temperature:

(2) $$\frac{1}{\emptyset_{Drug}}+\frac{1}{m}\times \frac{1}{\left(1-\emptyset_{Drug}\right)}-2\chi_{Drug-Pol}=0.$$

Estimation of the FH interaction parameter is commonly conducted based on the Hansen solubility parameter estimation, as detailed in Equation (3) [32]:

(3) $$\chi \approx 0.34+\frac{v_{Drug}}{RT}\times {\left(\delta_{Drug}-\delta_{Pol}\right)}^{2},$$

where *v* is the molar volume of the drug and *δ* is the solubility parameter of each entity.

Originally introduced by Hildebrand and Scott, solubility parameters were defined as the square root ratio of cohesive energy and molar volume [33]. The use of solubility parameters has received rising interest as a guide for the prediction of drug–polymer miscibility [34]. A common approach to estimate the solubility parameter is the use of group contributions tables based on the chemical structure of molecules. In the current project, solubility parameters were estimated via both Fedors and van Krevelen/Hoftyzer group contribution methods [35,36]. The latter implemented the split of solubility parameters into three contribution terms—dispersive, polar, and hydrogen bond forces. Equations (4) to (7) illustrate the calculations for each contributing term:

(4) $$\delta_{tot}=\sqrt{\delta_{d}{}^{2}+\delta_{p}{}^{2}+\delta_{h}{}^{2}},$$

(5) $$\delta_{d}=\frac{\Sigma_{i}Fdi}{\Sigma_{i}Vi},$$

(6) $$\delta_{p}=\frac{\sqrt{\Sigma_{i}Fpi^{2}}}{\Sigma_{i}Vi},$$

(7) $$\delta_{h}=\frac{\sqrt{\Sigma_{i}Ehi}}{\sqrt{\Sigma_{i}Vi}},$$

where *F_di_*, *Fp_i_*, *E_hi_*, and *V_i_* are the group contributions for dispersive, polar, H-bond forces, and molar volume, respectively. The calculation of the solubility parameter and the molar volume of the drug were evaluated using both Fedors and van Krevelen methodologies. The calculation of the solubility parameters of polymers is based on the chemical structure of the repeat unit. In the case of copolymers, the calculation includes the ratio of each repeat unit. The molar volume of polymer was calculated as the ratio of molecular weight to density. 

The miscibility of drug in different carriers can be estimated by their position in the Bagley diagram. A Bagley plot is a two-dimensional projection of combined parameter *δ_v_* as a function of *δ_h_*, as reported in Equation (8):

(8) $$\delta_{v}=\sqrt{\left(\delta_{d}{}^{2}+\delta_{p}{}^{2}\right)}.$$

The main principle of this theory is based on the well-known concept “like dissolves like” by comparing dispersive/polar and H-bonding potential of molecules.

#### 2.2.2. Manufacturing/Preparation Methods

• Preparation of stock solution

Stock solutions of drug and polymer were prepared in the binary solvent mixture of interest DCM/EtOH 2:1 (*v*/*v*) at 50 mg/mL. Drug-to-polymer ratios of 20:80, 40:60, 60:40, and 80:20 (*w*/*w*) were tested, and hence stock solutions were adjusted for each ratio.

• Film casting

  ○ At room temperature

The drug–polymer solution was magnetically stirred at 100 rpm during 15 min. The final volume was fixed at 5 mL in order to control film thickness. The solution was spread on a Teflon tape (21 cm × 15 cm) and a 15 cm diameter funnel (VWR, Heverlee, Belgium) was put on top of the casting solution. The solvent evaporation was performed at room temperature (RT) for 1 week. This allowed for similar evaporation rates for all productions while reaching quasi-equilibrium conditions. The slowest possible experimental evaporation rate represents the closest experimental approach to the thermodynamic models with evaporation to equilibrium. After 1 week, the film-casted ASD was removed from the Teflon plate and was stored in a vacuum oven for 48 h to remove residual solvent.

  ○ Under reduced pressure

A volume of 30 μL of drug–polymer solution was dropped into a standard aluminum DSC pan (TA Instruments, Leatherhead, UK). The pan was attached to a glass tube holder fixed on Syncore^®^ Polyvap basis (Büchi Labortechnik AG, Flawil, Switzerland). The Syncore^®^ Polyvap equipment enables the fast evaporation under vacuum of up to 96 samples in parallel. At 25 °C, pressure was gradually decreased to 5 mbar and maintained for 2 h to ensure solvent evaporation. After processing, the DSC pans were removed from the tube and then stored in a vacuum oven for 48 h. Each drug–polymer combination was produced five times. The miscibility of the generated films was investigated using mDSC (*n* = 3), while residual crystallinity was assessed by XRPD (*n* = 1). Finally, solvent content and degradation temperature were analyzed using TGA (*n* = 1). 

• Quench cooling

Quench cooling is a solvent-free screening test performed in situ DSC. Drug:polymer ratios of 20:80, 40:60, 60:40, and 80:20 (*w*/*w*) were generated in the DSC pan and analyzed in triplicate using a TA Instruments Q1000 calorimeter (TA Instruments, Leatherhead, UK). In DSC, a first heating step was applied on casted samples with a temperature up to 20 °C higher than the last thermal event (e.g., melting of the drug or the *T*_g_ of polymer). At the end of the first heating step, the mixture was in a molten state and a fast cooling temperature of up to −50 °C (−70 °C in case of IBU) was applied. Fast cooling rates limit the risk of drug recrystallization. Finally, a second heating rate was applied to analyze the phase behavior of the quench-cooled ASD generated in the DSC pan. Both heating steps were performed at a rate of 2 °C/min combined with a modulation of ±1 °C and a 40 s period.

• Atomization screening device 

**Figure 2:**
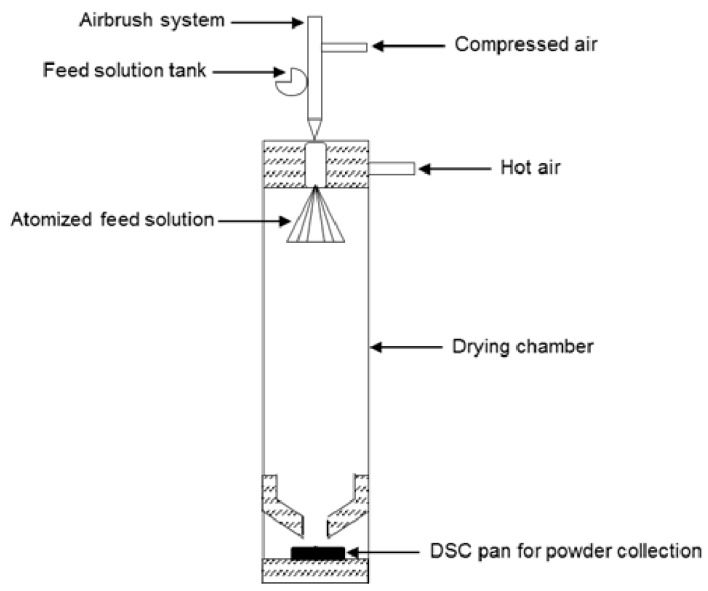
Schematic representation of atomization screening device. DSC: differential scanning calorimetry.

A volume of 1 mL of drug–polymer solution was atomized with a 0.15 mm Infinity airbrush (Harder & Steenbeck, Norderstedt, Germany). The air nozzle pressure was fixed at 1.5 bars. A bottom sawed glass tube of 15 cm (VWR, Heverlee, Belgium) was used as a drying chamber. A co-current airflow of 20 L/min at 100 °C was applied on top of the drying chamber with an Aoyue int 852 SMD rework station (Aoyue, Zhongshan, China). This ensured fast evaporation of the droplets. The outlet temperature of the chamber was measured at an average value of 40 °C ± 5 °C. After evaporation, powder was sent into a standard aluminum DSC pan (TA Instruments, Leatherhead, UK) to optimize powder collection and reduce powder handling. 3D-printed spare parts were designed to ensure sealing of the top of the drying chamber and to optimize powder collection from the drying gas (Figure 2).

• Spray dryer

Binary SDASDs were prepared using the lab-scale Büchi B290 mini spray dryer (Büchi Labortechnik AG, Flawil, Switzerland). The feed solution was pumped to the nozzle via a peristaltic pump at 4 mL/min. An atomizing airflow of 9 L/min was applied to a 0.7 mm bifluid nozzle to create a spray. The aspirator was set at 100% and maintained at 65 °C. The combination of process and formulation parameters (e.g., equipment configuration) led to a temperature at the bottom of the chamber of approximately 45 °C. Powder was collected and stored under vacuum at room temperature (RT) for 48 h. All productions were obtained with the spray dryer operating in a closed loop configuration.

#### 2.2.3. Analytical or Characterization Methods

• Modulated differential scanning calorimetry

mDSC analyses were carried out using a TA Instruments Q1000 calorimeter (TA Instruments, Leatherhead, UK). Inert atmosphere was maintained in the chamber purged with a 50 mL/min flow rate of dry nitrogen. A refrigerated cooling accessory (RCS90) was used to cool the samples during analysis. Data were processed using Universal Analysis 2000 software (TA Instruments, Leatherhead, UK). The temperature and enthalpy were calibrated using indium, while the heat capacity calibration was performed at 96.85 °C using sapphire disks. About 1.5–4 mg of sample were analyzed in triplicate (*n* = 3) using closed standard aluminum pans (TA Instruments, Leatherhead, UK). Samples were heated at a rate of 2 °C/min combined with a modulation of ±1 °C and a 40 s interval period. The chosen modulation parameters were validated using Lissajous figures. The inflection point temperature was used to report the *T*_g_ value. The *T*_g_ width was estimated as the distance between the onset and end of the transition region.

• X-ray powder diffraction

XRPD experiments were conducted on an X Bruker AXS D8 Advance (Bruker, Karlsruhe, Germany) at RT. A few milligrams of each sample was dropped on the center of a silicium monocrystal holder. Analyses were carried out over the range 4.5–30° at a scan speed of 2.5 s/step and a step size of 0.02°. Data obtained were processed and analyzed using Eva DIFFRAC-SUITE software (Bruker, Karlsruhe, Germany). In the integrated patterns, the intensity was displayed as a function of 2θ. 

• Thermogravimetric analysis

Experiments were conducted on TGA/SDTA 851e (Mettler Toledo, Columbus, OH, USA). The chamber was swept by a 50 mL/min flow rate of dry nitrogen. Data were processed using the Mettler Toledo software STARe (Mettler Toledo, Columbus, OH, USA). Experiments were carried out in 100 μL pierced aluminum crucibles. In parallel to mDSC procedure, the samples were heated from 25 °C to 300 °C at 2 °C/min. 

• Scanning electron microscopy

The morphology of the formulation was evaluated with scanning electron microscopy (SEM) using the Phenom Pro SEM (Phenom-World BV, Eindhoven, The Netherlands). Powder was placed at the center of an aluminum stud with a conductive double-sided carbon adhesive tape. Accelerating voltage and working distance of respectively 5 kV and 25–30 μm were applied during observations. Pictures were collected and edited using ProSuite software (Phenom-World BV, Eindhoven, The Netherlands).

• Miscibility-based classification 

The drug–polymer miscibility of each solid dispersion was evaluated and classified into three categories. Samples that only displayed a single *T*_g_ (between the *T*_g_ of pure drug and polymer) in the reverse heat flow of mDSC and a halo pattern in XRPD were classified as ideal amorphous glass solution (A). The presence of two *T*_g_s in reverse signal was specific for amorphous phase separated samples (AA)—these formulations contained one drug-rich phase and one polymer-rich phase. The ASDs are more prone to recrystallization during storage than the glass solution. Finally, samples where the presence of residual crystallinity was detected were isolated (AC). The presence of crystalline material can be attributed to an incomplete amorphization after processing. The main characteristics are the presence of the drug melting in total heat flow of mDSC and the presence of sharp peaks in XRPD. The presence of residual crystallinity can also be linked to the presence of recrystallization and melting events during mDSC analysis. The recrystallization process is a sign of physical instability in the formulation. More precisely, the carrier has a limited potential to stabilize the amorphous form of the drug at high DL. The exothermic recrystallization might be triggered by the low heating rate performed during analysis and/or by the presence of crystalline seeds formed in the formulation after processing. It has to be noted that mDSC is not able to detect domains smaller than 30 nm and to resolve two *T*_g_s if they are separated by a range smaller than 10 °C [37].

• Principal component analysis

PCA was performed using Simca 14.0 software (Umetrics, Malmö, Sweden). PCA analysis was used to explore the data compiled for the four drugs. This statistical analysis aimed to qualitatively compare the potential of the screening method to mimic the spray dryer results. The visual relative location of each predictive method from the spray dryer was evaluated using the PCA score plot. Raw data were mean centered and scaled to unit variance.

## 3. Results and Discussion

### 3.1. Theoretical Models Based on the Thermodynamics of Mixing

Solubility parameters have been extensively used as a guide for drug–polymer miscibility in many systems. The calculated values of solubility parameter and the molar volume of each drug and polymer—evaluated according to Fedors and van Krevelen methodologies—are depicted in Table 3.

The results displayed in Table 3 indicate that the *δ* value differs between the Fedors and van Krevelen methodology. Therefore, the calculation of the *δ* coefficient is method-dependent, and each compound has different *δ* values depending on the group contribution method used [36,38]. One reason is that each method calculates the cohesive energy and the molar volume differently, resulting in a deviation between method calculations. More specifically, the Fedors method is known to provide a less-accurate estimation of cohesive energy but a more precise value of molar volume [14].

This is mainly attributed to the larger data set in the Fedors method compared to other existing group contribution methods (e.g., van Krevelen). The absence of a double bond conjugated increment is one example of the limited data set provided by van Krevelen method [36]. This has no influence on the *δ* calculation of a simple molecule like IBU. However, *δ* calculations made for NAP and CAR are impacted by the insufficient level of information given by the van Krevelen group contribution method to evaluate complex aromatic structures. The use of the group contribution methods to estimate the *δ* coefficient is based on a thorough analysis of the chemical structure of compounds. Additionally, the estimation of *δ* can vary greatly in the case of hypromellose polymers. The properties of hypromellose polymers are defined by a large range of molecular weight and percentage levels of methoxyl, hydroxyl, and hydroxypropyl group substitutions on the cellulose chain [39]. This results in *δ* values being inaccurate when using the group contribution methods. For instance, a deviation in *δ* value will impact the results obtained for the Greenhalg criterion, FH parameter calculation, phase diagram, and Bagley plot construction (Equations (2), (3), and (8)).

The difference between drug and polymer solubility parameters (Δ*δ*)—usually called the Greenhalg criterion—was used to predict the drug–polymer miscibility. A difference value lower than 7 MPa^½^ between the two components indicates favorable mixing conditions [14]. Solubility parameter values derived from both Fedors and van Krevelen methods were used, and results are summarized in Table 4. 

All drug/polymer combinations—except IBU mixed with HP50—displayed absolute values of Δ*δ* lower than 7 MPa^½^, indicating that all systems would be completely miscible in all proportions. This result suggests that the selected polymers were good candidates for each model drug. A Δ*δ* value of 7.4 MPa^½^ was obtained for the combination of IBU and HP50, implying that some degree of immiscibility might appear depending on the drug:polymer ratio. The main reason why the Greenhalg criterion is predicting favorable mixing conditions among the selected compounds is the narrow range of polymer *δ* values (23.0–27.4 and 22.5–26.7 MPa^½^, respectively, obtained from Fedors and van Krevelen). Thus, evidence of potential incompatibilities between drug and carriers cannot be predicted by the Greenhalg criterion.

Furthermore, the FH parameter was evaluated from the solubility parameter approach, as detailed in Equation (3). Two sets of FH parameters were derived from Fedors and van Krevelen, respectively, and the *χ* values are reported in Table 4. As aforementioned, a low FH value reduces the free energy and hence favors mixing conditions. For example, the lowest value of ITR-FH coefficient was found when combining the drug with HP50 (0.4), while the highest value was found with EUD (2.4).

**Figure 3:**
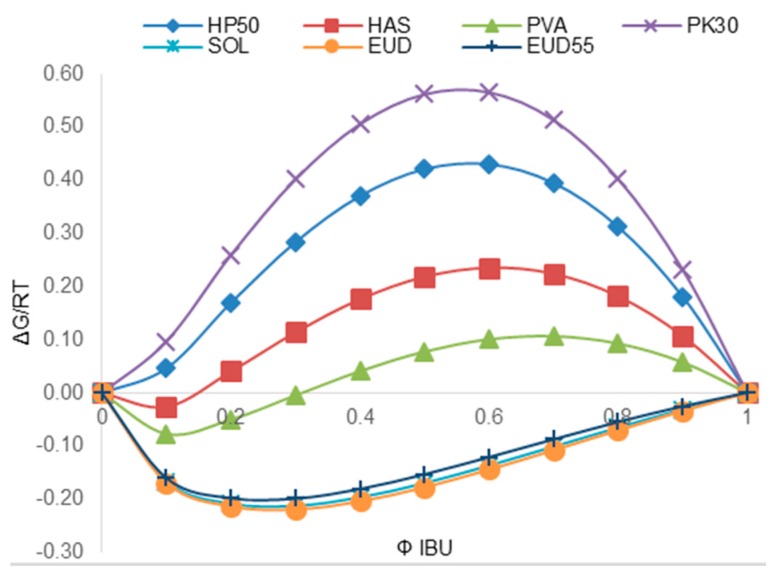
Phase diagram derived from *δ*(F) estimation at 25 °C for mixtures of IBU and PK30, HP50, HAS, PVA, EUD55, SOL, and EUD (from top to bottom).

According to Equation (1), the construction of a phase diagram provides an estimation of drug–polymer miscibility as a function of drug volume fraction. In the current study, phase diagrams were plotted based on the FH coefficient derived from both Fedors and van Krevelen *δ* values. The phase diagram of IBU obtained at 25 °C with the set of tested polymers is represented in Figure 3. 

Negative values of free energy were found at all drug volume fractions for the mixtures of IBU with SOL, EUD, and EUD55, respectively. These three profiles displayed a minimum free energy value at an IBU volume fraction of around 0.3. Below this IBU volume fraction, the system formed a miscible and thermodynamically stable glass mixture. Above 0.3, the system is in a metastable supersaturated state where phase separation might occur depending on the drug crystallization behavior and molecular mobility. Otherwise, the phase diagrams of IBU with HAS and PVA displayed a negative profile of Gibbs free energy up to a critical drug volume fraction where demixing occurred. Above this fraction, the free energy of mixing is positive. The system is unstable, and hence no thermodynamic barrier prevents the spontaneous destabilization of the system [17]. In this case, the presence of a single-phase system is limited, and varies depending on system composition. Finally, mixtures of IBU with HP50 and PK30 displayed a positive free energy at all proportions, indicating immiscibility between both components.

**Figure 4:**
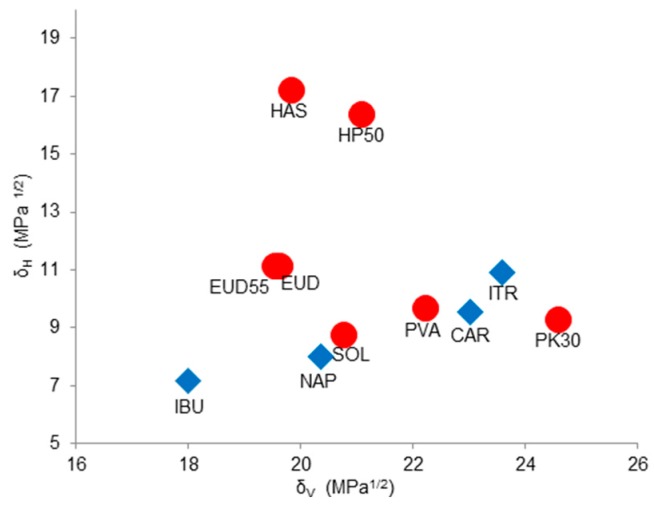
Specific locations of drugs (blue) and polymers (red) within Bagley diagram.

Drugs and polymers are represented in the Bagley diagram, as illustrated in Figure 4. The Euclidean distance was calculated between each point, and the results are provided in Table 4. Small drug–polymer distances represent the most promising associations. This is explained by the fact that both drug and polymer would have similar ability to interact via dispersive, polar, and/or H-bond forces [40]. 

As seen in Figure 4, the locations of PVA and PK30 are close to CAR and ITR. Similar *δ_v_* and *δ_h_* values were found between these entities, indicating favorable miscibility on the basis of weak polar interactions and H-bonding stabilization. This observation is confirmed by the smallest Euclidean distances obtained between those points when compared to other excipients. 

However, differences between the H-bonding donor and acceptor potential of molecules were not considered within the Bagley plot. Interestingly, the closest location of ITR in the diagram was assigned to PVA. Similar *δ_v_* and *δ_h_* values were found between these compounds, indicating the presence of favorable weak polar and H-bonding interactions. Nonetheless, both entities only contain H-bonding acceptor sites, and thus no H-bonding stabilization is expected. The conclusions delivered by the Bagley diagram must be confirmed in terms of chemical structure compatibilities. Stefanis et al. (2012) proposed an expanded solubility parameter approach where both acceptor and donor potentials were differentiated [41]. 

### 3.2. Comparison of Theoretical Models with Experimental Screening Methods with Regards to SDASD Development

#### 3.2.1. Evaluation of Drug–Polymer Miscibility

The ability of theoretical models, usual screening methods (film casting, quench cooling), and our novel atomization device to predict the phase behavior and therefore optimal DL of SDASDs was evaluated. ASDs were characterized systematically using mDSC and XRPD, and were subsequently classified using the miscibility-based classification described in the Methods chapter. Phase behavior differences were observed among the different experimental screening methods, and the influence of parameters causing miscibility differences of ASDs was investigated.

**Figure 5:**
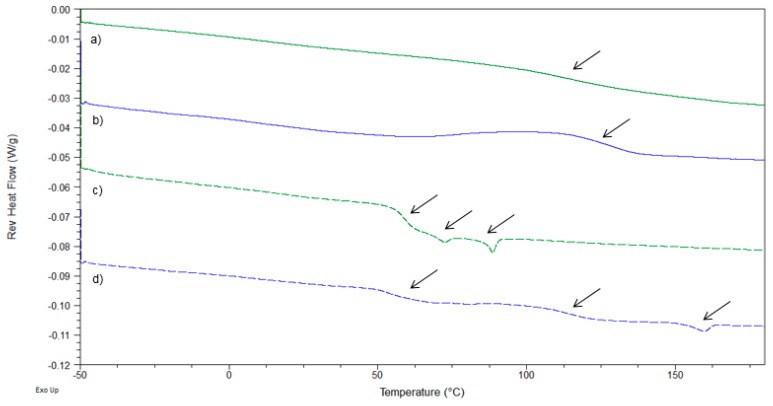
Reverse heat flow signals of ITR/PK30 40:60 (*w/w*) produced by (**a**) (green-solid line quench cooling) and (**b**) (blue-solid line) spray dryer. ITR/PK30 60:40 (*w*/*w*) produced by (**c**) (green-short dash) quench cooling and (**d**) (blue-short dash) spray dryer.

An illustration of how the miscibility-based classification was applied is depicted in Figure 5, showing the case of the ITR/PK30 ASD produced by quench cooling and spray drying at 40:60 and 60:40 (*w*/*w*), respectively.

**Figure 6:**
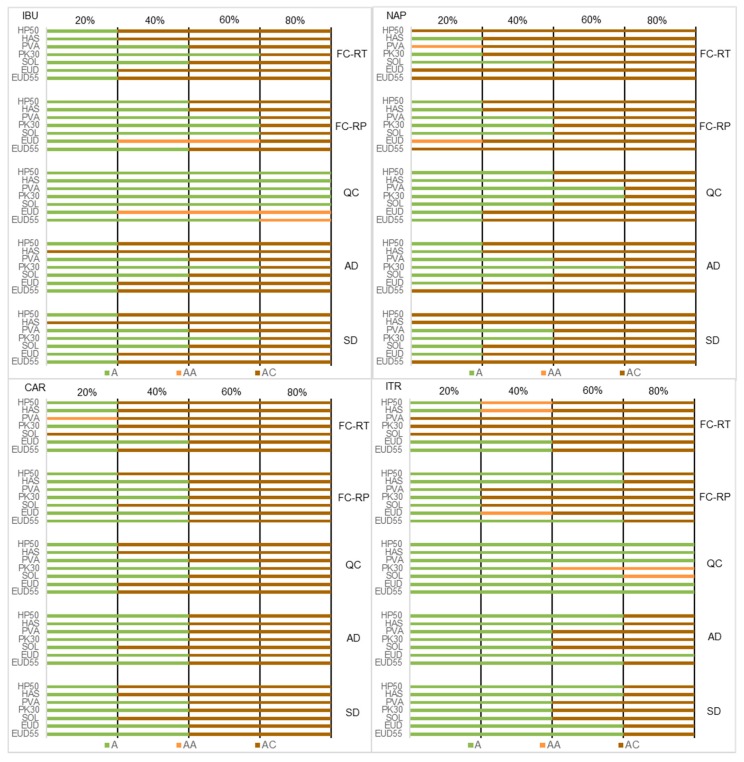
Phase behavior of IBU, NAP, CAR, and ITR amorphous solid dispersions (ASDs) (from top to bottom) produced by film casting (FC) performed: at RT (FC-RT) and under reduced pressure (FC-RP); (QC) quench cooling, (AD) atomization screening device, and (SD) spray dryer as a function of drug loading (DL) for the set of polymers tested. A: ideal amorphous glass solution; AA: amorphous phase-separated sample; AC: sample with the presence of residual crystallinity.

Both quench-cooled and SDASD of ITR/PK30 40:60 (*w*/*w*) displayed a single *T*_g_ in reverse heat flow. No drug melting endotherm was detected in total heat flow (data not shown). The *T*_g_ value of the ASD was found to be between the *T*_g_ of the pure drug and the pure polymer. In addition, no crystalline peaks were detected in XRPD. Thus, both 40:60 (*w*/*w*) ASDs were classified as amorphous glass solution (A). The reverse heat flow signal of the quench-cooled solid dispersion of ITR/PK30 at 60:40 (*w*/*w*) indicated the presence of a single *T*_g_ value at 58.9 °C and two endothermic events at 72.1 °C and 88.4 °C. The *T*_g_ corresponds to the glassy state of pure ITR. Both endothermic events are liquid crystal states called mesophases, inherently linked to pure glassy ITR [42]. XRPD analysis confirmed the absence of residual crystallinity. This sample was categorized as a phase-separated amorphous system (AA) despite the absence of a second polymer *T*_g_ in the thermogram. Finally, the thermogram obtained for spray-dried ITR/PK30 at 60:40 (*w*/*w*) displayed the *T*_g_ of pure glassy ITR, mesophases endotherms, and the melting endotherm in both reverse and total heat flow. No crystalline peaks were detected in XRPD. However, the presence of a broad recrystallization event was detected in the baseline of total heat flow. Herein, the polymer stabilization was limited, and potential crystalline seeds may have formed. This system was metastable and tended to recrystallize when heated (AC). Overall results obtained for IBU, NAP, CAR, and ITR formulations are summarized in Figure 6.

The use of a solvent-free screening method—namely quench cooling—resulted in the highest degree of miscibility compared to other techniques, especially spray drying. As seen in Figure 6, glass solutions of IBU and ITR were produced up to 80% (*w*/*w*) with five different carriers by quench cooling. When considering the SDASDs, a glass solution of IBU was obtained with 40% (*w*/*w*) PK30 only. Moreover, four glass solutions of ITR were achieved at 60% (*w*/*w*) DL for all investigated polymers. This study revealed miscibility differences of ASDs obtained by both solvent and solvent-free preparation methods. The high degree of miscibility achieved by quench-cooled ASD is particularly true for GFA class III drugs such as IBU and ITR that have low tendencies for crystallization. In such a case, a very low amount of polymer is needed to stabilize the amorphous form of the drug. The miscibility level of quench-cooled ASDs of IBU and ITR was superior to NAP and CAR samples due to the highest inherent stability tendency of the pure amorphous drug in molten state (Table 1) [29]. 

**Figure 7:**
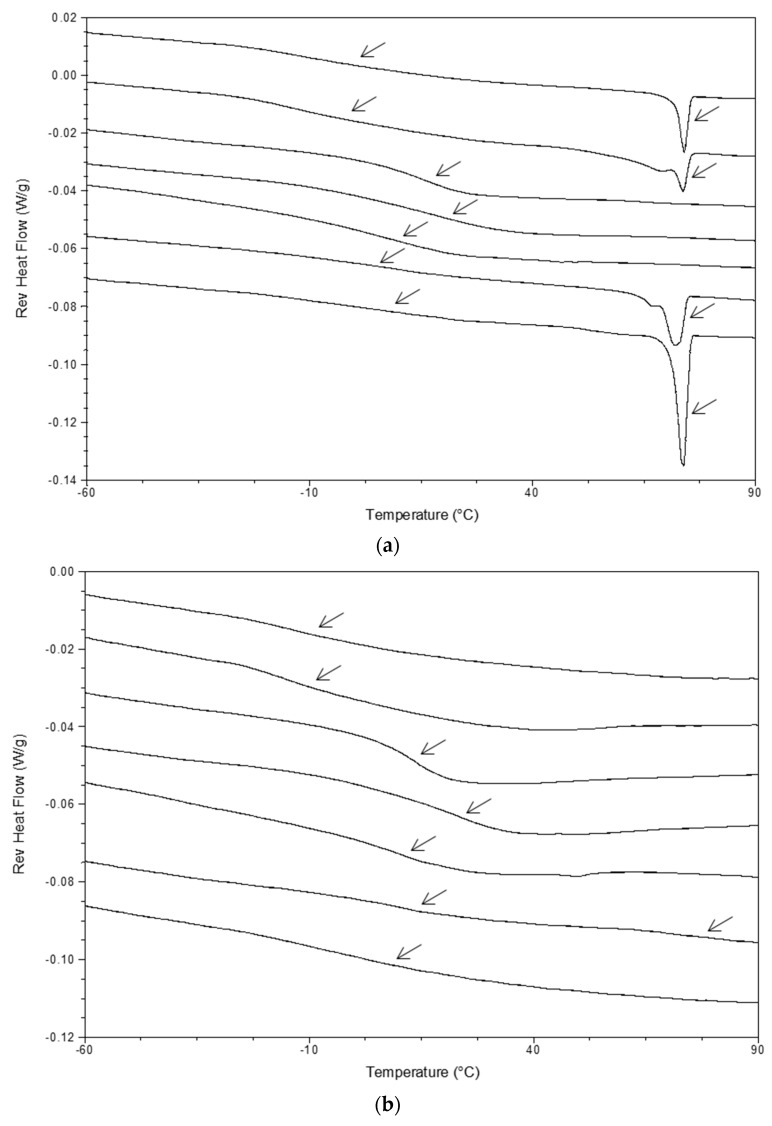

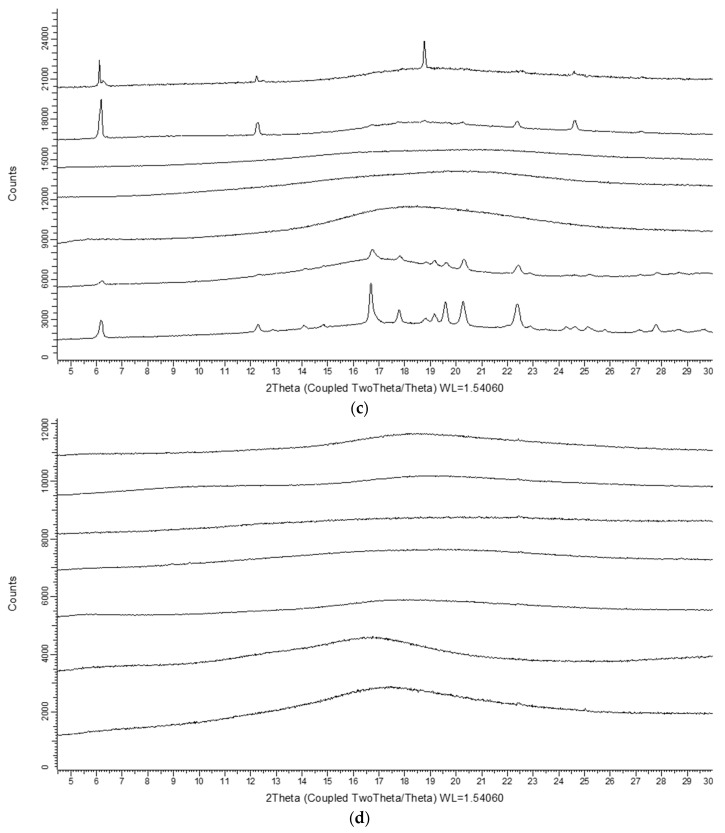
(**a**,**b**) Reverse heat flow signals and (**c**,**d**) X-ray powder diffraction (XRPD) patterns of ASDs of IBU 40:60 (*w*/*w*) with HP50, HAS, PVA, PK30, SOL, EUD, and EUD55 (from **top** to **bottom**) produced by film casting performed: (**a**,**c**) at RT and (**b**,**d**) under reduced pressure.

The influence of solvent evaporation rate was evaluated when comparing the miscibility of film-casted ASDs produced at RT and at low pressure in Figure 6. Among the set of tested drugs, increasing solvent evaporation kinetics was found to improve the drug–polymer miscibility. This observation was found particularly in the thermograms of film-casted IBU ASD 40:60 (*w*/*w*) produced at RT and under reduced pressure, shown in Figure 7. Film-casted IBU ASDs 40:60 (*w*/*w*) with PVA, PK30, and SOL formed a glass solution after 1 week evaporation at RT. These formulations displayed a single *T*_g_ in reverse heat flow and a large halo in XRPD. Nevertheless, residual crystallinity was detected by the presence of the drug melting endotherm in mDSC and sharp peaks in XRPD in the case of HP50, HAS, EUD, and EUD55 formulations. On the contrary, the production of film-casted IBU ASD 40:60 (*w*/*w*) at low pressure generated glass solutions with the different polymers, except for EUD ASD, where an amorphous phase-separated system was identified.

Mechanisms of phase separation followed by drug recrystallization are favored by a slow solvent evaporation rate [28]. This explains the limited degree of miscibility obtained for film-casted ASD produced at RT. The 1-week solvent evaporation test is not representative of real-time conditions, but was chosen in order to evaluate the ability of polymers to stabilize the amorphous form of the drug under unfavorable solvent evaporation conditions. As shown in previous studies, the presence of potential interactions between H-bond acceptor polymers PVA and PK30 and H-bond donor drugs IBU and NAP frequently resulted in the stabilization of the amorphous form of the drug [11,43]. In the current study, the list of polymers that stabilized the amorphous form of the drug during the slow evaporation process generally provided SDASDs at higher DL. On the contrary, fast solvent removal at low pressure increases the degree of supersaturation and favors the formation of a kinetically-trapped amorphous system.

Interestingly, this study found that the spray drying did not necessarily generate the highest level of miscibility. Despite an evaporation speed less than 1 s (compared to minutes and hours in the case of film casting performed at reduced pressure and at RT), residual crystallinity was detected in the case of SDASDs of IBU and NAP/HAS 20:80 (*w*/*w*) (Figure 6). On the contrary, the respective film-casted ASDs produced at RT and at low pressure were fully amorphous. In conclusion, evaporation rate does not seem to be the only factor that governs drug–polymer miscibility. Additional criteria such as specific surface area and surface properties of film compared to spray-dried powder might be of interest, and would need more investigation.

**Figure 8:**
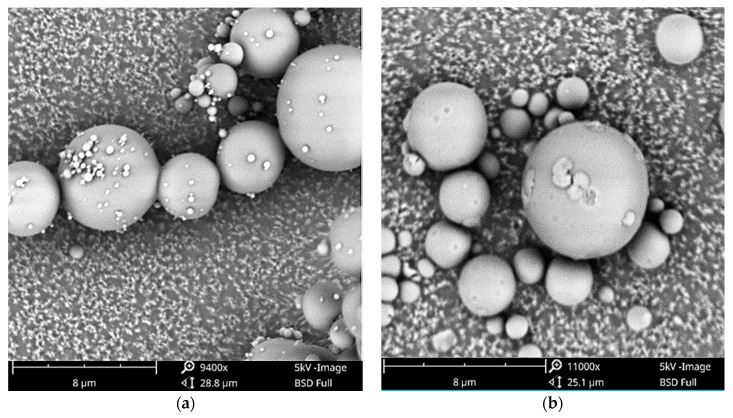
Scanning electron microscope (SEM) observations of NAP/PVA 40:60 (*w*/*w*) produced by (**a**) atomization device and (**b**) spray dryer.

Throughout the entire set of tested drugs, miscibility prediction accuracy of the atomization device was superior with regard to spray-dried formulations compared to the other screening methods (Figure 6). However, the degree of miscibility of samples prepared by the atomization device was higher—except for IBU ASDs—compared to the SDASDs (Figure 6). For instance, glass solutions of ITR/EUD were obtained up to a maximum DL of 60% and 80% (*w*/*w*) for the spray dryer and atomization device, respectively. Worku et al. (2014) recently investigated the drug–polymer miscibility across a spray dryer [44]. The authors found that different droplet sizes (and hence particles) would generate differences in phase behavior. One explanation was that each droplet undergoes different micro-environment drying conditions, resulting in phase behavior differences. In the current study, the design of the atomization device did not include a cyclone to separate powder from the drying gas. Airflow and powder were directly sent into an aluminum DSC pan where powder was collected and airflow was evacuated through the bottom of the chamber. To confirm this hypothesis, SEM observations were performed on the NAP/PVA (40:60) (*w*/*w*) ASD (Figure 8). The analysis revealed that powders generated by the atomization device had a smaller particle size distribution than the spray-dried samples. This reinforces the potential dependence of drug–polymer miscibility with respect to the inherent particle size of generated powders, and suggests that particle size and specific surface area are potential critical formulation attributes.

**Figure 9:**
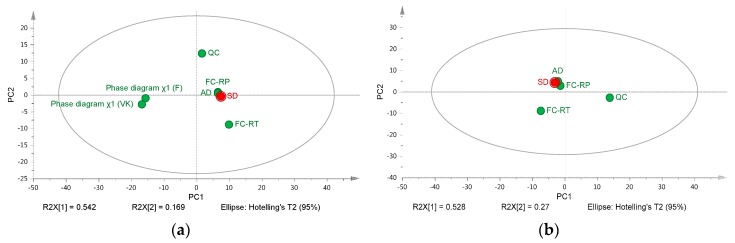
(**a**) Principal component analysis (PCA) score plots analysis of drug–polymer miscibility predicted by thermodynamic models and obtained for ASDs produced by film casting performed: at RT (FC-RT) and under reduced pressure (FC-RP); (QC) quench cooling, (AD) atomization screening device, and (SD) spray dryer for IBU, NAP, CAR, and ITR mixed with the set of polymers tested; (**b**) The PCA was repeated excluding the thermodynamic models from the comparison.

PCA is a suitable statistical method used to assess variability; i.e., statistical information over a large dataset by reducing the number of variables [45]. In this case, PCA analysis was conducted as a visualization method to ease the comparison of several screening methods and evaluate their prediction accuracy in terms of miscibility with regards to SDASDs. The miscibility classes of ASD established in Figure 6 and the results derived from the phase diagrams plotted at 25 °C were included as variables in the PCA analysis. PCA analysis was compiled for the four drugs and each manufacturing method; results are depicted in Figure 9a. Two principal components were sufficient to explain 71.1% of the miscibility classes’ variability. 

The miscibility prediction given by the thermodynamic models was found to be not in good alignment with experimental screening methodologies and spray-dried formulations. The miscibility differences found between thermodynamic models and experimental results revealed a gap that could be explained by thermodynamic and kinetic miscibility. Formulations produced by spray drying generally formed a kinetically stable state over the solubility and miscibility curves of crystalline and amorphous drug in carrier [22]. The limitations of FH theory to predict the mixing of pharmaceutical ASDs have already been reported in the literature [46]. Efforts to implement models that better integrate the hydrogen bonding contribution in solid dispersion systems is essential for the prediction of drug–polymer miscibility [47]. Recently, the PC-SAFT model was found to be a suitable approach for modelling the phase behavior and long-term stability of drug/polymer blends [48]. However, the influence of the preparation method, the distribution of the drug in the carrier, the presence of a ternary system (solvent), and directional interactions between the drug and the carrier are relevant factors that are not consistently considered in the theory of mixing. This study confirmed the limitations of those theoretical considerations based on the theory of mixing and demonstrated that experimental screening methods (kinetic considerations) are required to predict the phase behavior of SDASDs. 

Interestingly, PCA projections of experimental methods were located in the same region. The PCA score plot of Figure 9b is the graphical representation of the same PCA analysis as before, excluding the thermodynamic models. In this case, the two principal components were sufficient to explain 79.8% of the miscibility classes’ variability among the experimental screening methods. The results indicated that the miscibility prediction given by quench cooling and film casting at RT deviated from SDASD location. As discussed previously in Figure 6, this can be explained by the high degree of miscibility reached by quench cooling in the case of GFA class III drugs and the limited miscibility of film-casted samples prepared at RT. Drug–polymer miscibility predicted by atomization screening device was found most predictive for SDASDs by PCA, confirming previous results (Figure 6).

Results obtained in this section highlighted how process conditions and parameters of the different screening methods impact the phase behavior of ASDs: solvent evaporation rate was identified as a critical process parameter that explains miscibility variability among the differently produced solid dispersions and tested screening methods. Increase of solvent evaporation rate was found to improve drug–polymer miscibility of ASDs [49], explaining to a large extent the observed miscibility variability of samples prepared by film casting at RT and spray drying, respectively. Indeed, slow solvent evaporation favors drug crystallization and hence represents unfavorable conditions for the preparation of robust ASDs [28]. On the contrary, the operating mode of the atomization device results in a fine atomization of the feed solution followed by a fast solvent evaporation, being comparable to a regular spray dryer. These conditions reduce the time window for drug recrystallization, and are suitable for the production of robust solid dispersions with a high DL. Interestingly, the highest degree of miscibility was obtained by quench cooling. In this specific case, the GFA/GS of pure amorphous drug and the speed of cooling rate when processing (not investigated in this study) were the main critical factors to prevent drug crystallization.

As aforementioned, the limited miscibility of some SDASDs compared to film-casted samples (e.g., observed for IBU and NAP/HAS 20:80 (*w*/*w*)) suggests that additional formulation attributes influence the drug–polymer miscibility. This can be explained on the basis that film and particle productions generate differences with regard to specific surface area, surface properties, and particle size distribution. The ability of the atomization device to replicate the mechanism of particle formation makes this system superior to film-casted samples where significant differences in particle size and surface properties are expected compared to spray-dried specimens. In conclusion, due to its improved ability to predict the phase behavior of SDASDs compared to conventional approaches, our atomization device would allow the prediction of the performance of screened formulations and determination of optimal DL for spray-dried dispersions in the early phase of drug development.

#### 3.2.2. Evaluation of Glass Solution Thermal Properties

The thermal properties of amorphous material affect the performance of solid dispersions. *T*_g_ value and width are considered as an indicator of ASD physical stability and homogeneity. Samples with low *T*_g_ value and/or large *T*_g_ width are assumed to be more prone to phase separation and drug recrystallization [50]. 

**Figure 10:**
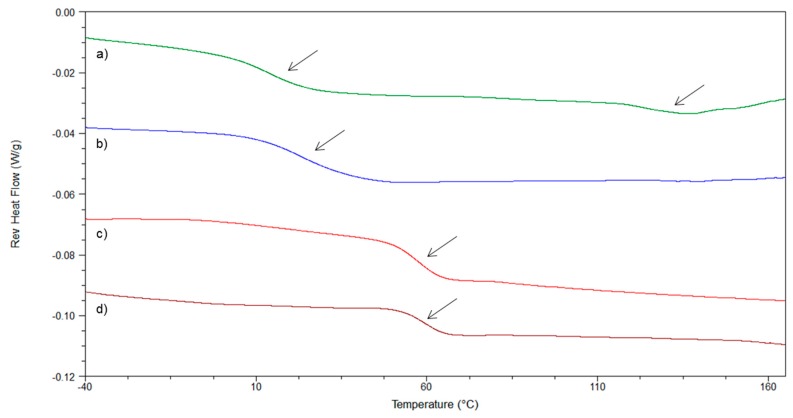
Reverse heat flow signals of NAP/PVA 40:60 (*w*/*w*) produced by (**a**) (green-solid line) film casting at RT; (**b**) (blue-solid line) film casting at low pressure; (**c**) (red-solid line) atomization device; and (**d**) (brown-solid line) spray dryer.

As a case study, the reverse heat flow signals of NAP/PVA 40:60 (*w*/*w*) ASD produced by film casting at RT and under vacuum by atomization device and spray drying are given in Figure 10. The thermogram of film-casted ASD produced at RT indicates the presence of a mixed *T*_g_, and the drug melting endotherm reveals the presence of residual crystallinity. The reverse heat flow signals of all other formulations displayed a single *T*_g_, characteristic of a glass solution. However, among the samples whereby glass solutions were produced, different thermal properties are clearly identified. This would probably result in different stability performance upon storage. 

Basically, the film-casted glass solution prepared under reduced pressure showed a lower *T*_g_ value and a broader *T*_g_ width compared to spray-dried sample properties. One possible cause is the presence of residual solvent acting as a plasticizing agent and decreasing the *T*_g_ value. This will increase the molecular mobility and hence favor the drug crystallization. Furthermore, the drying kinetics would probably interfere in the formation of a drug–polymer gradient during the process, and hence impact the final distribution of the drug in the carrier [27]. Paudel et al. (2013) highlighted the effect of inlet temperature and atomizing airflow of spray drying on the *T*_g_ value and width of NAP/PVPK25 ASD [51]. In the present case, the benefit of the atomization screening device is to provide glass solutions with similar thermal properties to SDASDs.

**Figure 11:**
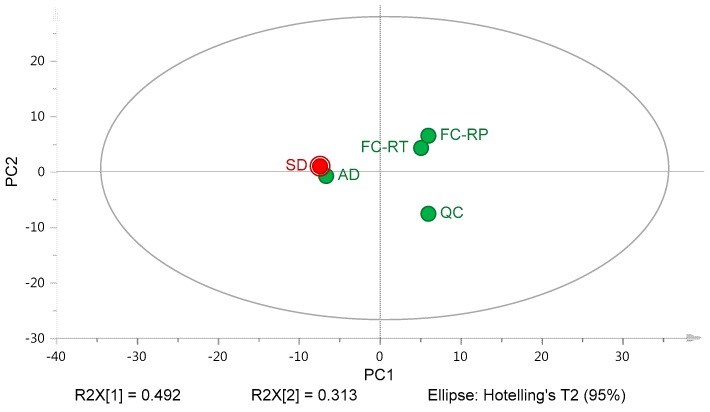
PCA score plot analysis of *T*_g_ values and widths of glass solutions produced by film casting performed: at RT (FC-RT) and under reduced pressure (FC-RP); (QC) quench cooling, (AD) atomization screening device, and (SD) spray dryer for IBU, NAP, CAR, and ITR mixed with the set of polymers tested.

The average *T*_g_ values and widths of glass solutions obtained during mDSC analysis (*n* = 3) were used as variables in a PCA analysis. The PCA score plot displayed in Figure 11 summarizes the differences of thermal properties of glass solutions (in terms of *T*_g_ value and width) produced by all experimental methods for the four model drugs. Two principal components were found to explain 80.5% of the solid-state properties’ variability. 

According to the PCA score plot, three different areas were identified. The glass solutions prepared by film casting techniques and solvent-free screening (namely quench cooling) formed two distinct clusters, both positioned further away from the spray-dried glass solution. More specifically, the presence of three distinct clusters in the PCA score plot highlights the gap between film-made ASD and powder produced after atomization and quick solvent evaporation. The results derived from PCA analyses (Figure 9 and Figure 11) demonstrated the poor capacity of film-based screening to predict the phase behavior and the thermal properties of SDASDs. At this stage, the determination of the optimal DL would require additional tests. Finally, the thermal properties (*T*_g_ value and width) of glass solutions prepared by atomization device had the closest position to spray-dried glass solutions. The results obtained using PCA confirmed the observations revealed by the thermograms discussed above in Figure 10. This observation revealed that the glass solutions produced by atomization device had similar thermal properties (in terms of *T*_g_ value and width) to spray-dried glass solutions, and offer the highest prediction with regards to SDASDs. Thus, the above observations indicated that the solid state of ASDs—and more specifically glass solutions—are influenced by the manufacturing method. Based on the aforementioned considerations, the atomization device was designed and developed in order to mimic the spray dryer process and to produce particulate specimens at small scale. Based on these considerations, the ability of our miniaturized screening to predict the *T*_g_ properties (value and width) would provide an indication of the physical stability of SDASDs, and therefore fine-tune the carrier selection during screening stages.

#### 3.2.3. Evaluation of Polymer Ranking

The first goal of any screening method is the selection of the right polymer for the right drug. The ranking of the three promising polymers provided by each of the predictive methods is depicted in Table 5. More precisely, the polymer ranking regarding Greenhalg criterion, Bagley plot, and FH theory was independently established based upon the value obtained for Δ*δ*, Euclidean distance, and FH coefficient, respectively. The polymer ranking of each manufacturing method was determined by evaluating the polymer’s potential to provide glass solutions at high DL. The average *T*_g_ value and width were also considered. The prediction accuracy of each screening approach was evaluated by calculating the probability of predicting the polymer type (*f*1) and the polymer rank order (*f*2) of SDASDs (Table 5).

The results obtained for thermodynamic models clearly indicate that over the range of tested polymers, the theoretical approach could not predict the most suitable carriers used for SDASDs. This was shown by the *f*1 and *f*2 values obtained for the four model drugs. For instance, EUD and EUD55 were found to be the best polymers to stabilize ITR up to 60% (*w*/*w*) after spray drying (Table 4). However, no thermodynamic model was able to selectively predict the polymers. In addition, prediction discrepancies were found among the different thermodynamic approaches, as reported in previous sections. This was notably observed between Fedors- and van Krevelen-based models.

Quench cooling was generally able to predict the first two polymers that produced glass solutions at high DL with spray drying (Table 5). However, differences were obtained, especially for the third polymer. According to the polymer ranking provided by the melt method, HAS and SOL were ranked in third position for IBU and CAR. However, ASDs prepared by spray drying identified SOL and EUD55 as third carrier choice. The results contained in Table 5 demonstrate that the ranking of polymers differed between the quench cooling and the spray drying approaches. Thus, it can be assumed that different polymers would be selected for the development of ASDs by solvent or melt process. The main drawback of quench cooling is the lack of selectivity among the different tested polymers. This observation can be explained by the fact that a high degree of miscibility is achieved by quench cooling in general.

The film casting techniques provided disparate results regarding the prediction accuracy of suitable polymers for SDASD development (Table 5). The film casting performed at RT predicted the exact polymer rank order for IBU SDASDs. However, low probability values were determined with regards to the prediction of excipients involved in NAP ASDs. Furthermore, the atomization device displayed the highest probability values for all four model drugs.

**Figure 12:**
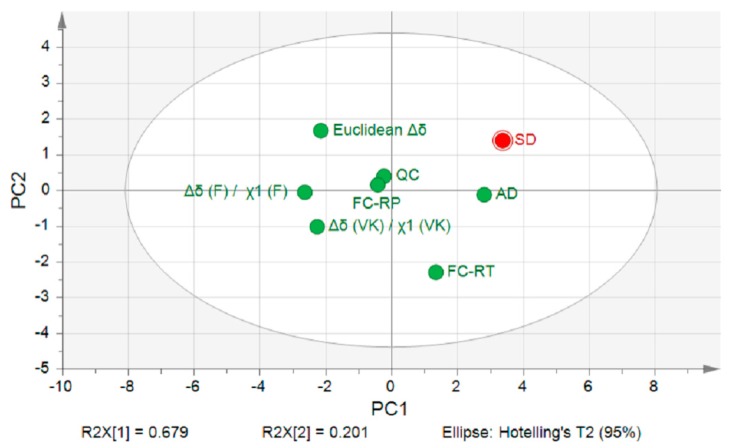
PCA score plot analysis of polymer rank order for thermodynamic models, film casting performed: at RT (FC-RT) and under reduced pressure (FC-RP); (QC) quench cooling, (AD) atomization screening device, and (SD) spray dryer for IBU, NAP, CAR, and ITR with the set of polymers tested.

A PCA analysis based on the values obtained for *f*1 and *f*2 was conducted to evaluate the prediction accuracy of each screening method to predict the polymer rank order with regards to SDASDs (Figure 12). The two principal components used in PCA explained 88.0% of the polymer ranking variability. 

According to the PCA score plot, the locations of polymer ranking provided by thermodynamic models were found to be opposite to the spray dryer point, which is in compliance with previous observations. Among the standard screening methods, the location of polymer ranking predicted by film casting performed at RT was closer to the spray dryer ranking than both quench cooling and film casting under reduced pressure. Increasing the solvent evaporation rate of film casting—hence trying to mimic the spray dryer conditions—did not improve the polymer ranking prediction. Among the standard screening methodologies, film casting performed at RT was found to be the best fit regarding polymer selection for SDASDs. In this case, the screening strategy reflects the ability of the carrier to inhibit drug crystallization under unfavorable process conditions (slow solvent evaporation). However, the miscibility differences found between film casting at RT and spray drying (Figure 6) does not allow the selection of an optimal DL at this stage of formulation development, and would require additional tests. Furthermore, the PCA analysis revealed that the polymer ranking predicted by the atomization device was the closest in location with respect to spray dryer results. This confirmed the observations made in the case of IBU, CAR, and ITR, where the atomization screening device and spray dryer provided the same polymer ranking for the entire set of tested carriers (Table 5). Polymer ranking differences between atomization device and spray dryer are mostly explained by the PC2 axis. According to the loadings graph (data not shown), the PC2 axis is mainly linked to *f*1 and *f*2 values obtained for NAP ASDs.

Thus, the use of PCA allowed a visual differentiation and comparison between the screening methodologies and spray drying. PCA analysis highlighted the miscibility differences of ASDs prepared by different screening/manufacturing methods on the basis of a high number of variables/drug–polymer combinations. Based on the PCA results, the novel atomization screening device was found to be the most suitable optimal screening method to predict the drug–polymer miscibility and the solid-state properties of SDASDs (Figure 9 and Figure 11). This indicates the superior ability of the atomization screening device to predict both the right polymer and the optimal DL for SDASD development compared to standard screening methods. This observation results in many advantages compared to usual screening approaches, such as reducing project timelines and improving the transition and scale-up from early stage to the first batch productions. The novel atomization device allows the production of spray-dried powder at milligram scale with a very fast solvent evaporation of the feed solution, correlating well with process conditions occurring during regular spray drying. The technique allowed identifying and understanding in a very comprehensive manner both critical process parameters (e.g., solvent evaporation rate) and critical material attributes of the spray-dried powder (e.g., particle size and surface properties). In addition, a complementary polymer ranking based on in vitro dissolution profiles would be of interest to investigate the dissolution performance of the most promising glass solutions discovered during screening phases. This would help to gain further insight into potential bioavailability enhancement of selected ASDs.

## 4. Conclusions

This study highlighted the complexity of the drug–polymer miscibility prediction. The phase behavior of ASD is influenced by formulation and process factors such as polymer choice, DL, solvent/solvent-free preparation method, solvent evaporation rate, and final nature of product (mainly film or powder). PCA analysis revealed that our novel atomization device provided a much better prediction of drug–polymer miscibility, glass solutions properties, and polymer selection of SDASDs than classic film casting and quench cooling techniques. The prediction accuracy of screening methodologies for drug–polymer miscibility of SDASD formulations was ranked as: atomization device > film casting under reduced pressure >> film casting at room temperature >> quench cooling >> thermodynamic models. Furthermore, the ability of screening approaches to select the most suitable carriers for robust SDASD development was ranked in the following order: atomization device >> film casting at room temperature >> quench cooling > film casting under reduced pressure >> thermodynamic models. 

Poor correlation was found between thermodynamic models based on theory of mixing and experimental results for the set of tested drug/polymer blends. Due to the limited ability of conventional screening approaches (solvent casting and quench cooling) to predict the phase behavior and thermal properties of SDASDs, appropriate selection of carrier and DL was not consistently guaranteed. 

Additionally, thermal and solid-state analyses revealed that critical process parameters (e.g., solvent evaporation rate) and critical material attributes of the spray-dried powder (e.g., particle size and surface properties) can impact the drug–polymer miscibility of solid dispersions. Our novel atomization system can better reproduce similar operating mode of a regular spray dryer than standard screening approaches, and therefore offers a much more robust and efficient alternative in ASD screening with regards to drug formulation development. Based on these considerations, our novel atomization device was found to better select adequate polymer and optimal DL in the early phases of drug development where usual screening methods have failed. Overall results obtained with our novel atomization device have emphasized its superior ability to reduce timelines and risk decision during a project, and correlate the information generated during the preformulation screening stage (where only milligrams of material is involved) with the final properties and performance of solid dispersion produced at large scale. 

## Figures and Tables

**Table 1:** Physico-chemical properties of ibuprofen, naproxen, carbamazepine, and itraconazole. GFA: glass forming ability; GS: glass stability.

Drug	Abbreviation	M_w_ (g/mol)	logP	H-Bond Donor Sites	H-Bond Acceptor Sites	*T*_g_ (°C)	*T*_m_ (°C)	GFA/GS
Ibuprofen	IBU	206	3.97	1	2	−44	76	III
Naproxen	NAP	230	3.18	1	3	6	158	I
Carbamazepine	CAR	236	2.45	1	1	50	177 (III)–193 (I)	I
Itraconazole	ITR	705	5.66	0	9	59	168	III

**Table 2:** Physico-chemical properties of selected polymers. HPMCAS-LF: hydroxypropylmethylcellulose acetate succinate fine grade; HPMCP: hydroxypropylmethylcellulose phthalate; PVPK30: polyvinylpyrrolidone; PVPVA: copolymer of *N*-vinyl-2-pyrrolidone and vinyl acetate.

Polymer	Abbreviation	*M*_w_ (g/mol)	True Density (g/cm^3^)	Dissolution pH	*T*_g_ (°C)	T Degradation (°C)
HPMCP-HP50	HP50	78,000	1.82	>5.0	140	160
HPMCAS-LF	HAS	18,167	1.29	>5.5	122	170
PVPVA	PVA	57,500	1.27	-	112	215
PVPK30	PK30	50,000	1.18	-	162	>300
Soluplus	SOL	115,000	1.03	-	80	190
Eudragit L100	EUD	125,000	1.28	>6.0	192	165
Eudragit L100-55	EUD55	320,000	1.25	>5.5	122	165

**Table 3:** Calculated three-dimensional solubility parameters (*δ*) and molar volume (*v*) for selected drugs and polymers according to Fedors (F) and van Krevelen (VK) tables.

	MPa^½^					cm^3^ mol^−1^	
	*δ*(F)	δ(VK)	*δd*(VK)	*δp*(VK)	*δh*(VK)	*v*(F)	*v*(VK)
IBU	20.9	19.4	17.9	2.2	7.2	195.5	195.5
NAP	23.4	21.9	20.1	3.0	8.0	178.3	203.1
CAR	28.1	24.9	22.0	6.6	9.6	154.1	168.8
ITR	26.6	26.0	22.8	6.0	10.9	403.6	434.5
HP50 ^a^	26.8	26.7	20.8	3.8	16.4	384.5	384.5
HAS ^a^	25.9	26.3	19.4	4.3	17.2	344.5	344.5
PK30	27.4	26.3	20.4	13.7	9.3	71.7	81.2
PVA ^b^	25.1	24.4	19.2	11.2	9.7	69.7	75.4
SOL ^c^	23.1	22.6	18.6	9.2	8.7	83.9	89.3
EUD ^d^	23.0	22.6	18.5	6.6	11.1	70.4	70.4
EUD55 ^d^	23.3	22.5	18.4	6.6	11.1	70.8	70.8

^a^: Values calculated based upon the range of substituent proportion on glucose ring found in supplier literature. ^b^: Block copolymer: *n* = 0.6 and *m* = 0.4. ^c^: Block copolymer: *n* = 0.57, *m* = 0.3, and *l* = 0.13. ^d^: Block copolymer: *n* = 0.5 and *m* = 0.5. CAR: carbamazepine; IBU: ibuprofen; ITR: itraconazole; NAP: naproxen.

**Table 4:** Summary of thermodynamic model calculations: solubility parameters differences Δ*δ* (namely Greenhalg criterion), Flory–Huggins (FH) coefficient (*χ*) both calculated according to Fedors (F), van Krevelen (VK) tables and Euclidean distance (EUC-d) calculated from Bagley plot.

Drug	Thermodynamic Model	HP50	HAS	PV30	PVA	SOL	EUD	EUD55
**IBU**	Δ*δ*(F)	5.9	5.0	6.5	4.2	2.2	2.1	2.4
	Δ*δ*(VK)	7.4	6.9	6.9	5.0	3.2	3.2	3.1
	*χ*(F)	3.1	2.3	3.6	1.7	0.7	0.7	0.8
	*χ*(VK)	4.6	4.1	4.1	2.3	1.2	1.2	1.1
	EUC-d	9.8	10.2	6.9	4.9	3.2	4.3	4.3
**NAP**	Δ*δ*(F)	3.4	2.5	4.0	1.7	0.3	0.4	0.1
	Δ*δ*(VK)	4.9	4.4	4.4	2.5	0.7	0.7	0.6
	*χ*(F)	1.2	0.8	1.5	0.6	0.4	0.4	0.3
	*χ*(VK)	2.3	1.9	1.9	0.9	0.4	0.4	0.4
	EUC-d	8.4	9.2	4.4	2.5	0.8	3.2	3.2
**CAR**	Δ*δ*(F)	1.3	2.2	0.7	2.9	5.0	5.1	4.8
	Δ*δ*(VK)	1.8	1.3	1.4	0.5	2.3	2.3	2.4
	*χ*(F)	0.4	0.6	0.4	0.9	1.9	1.9	1.8
	*χ*(VK)	0.6	0.5	0.5	0.4	0.7	0.7	0.7
	EUC-d	7.1	8.3	1.6	0.8	2.4	3.8	3.8
**ITR**	Δ*δ*(F)	0.3	0.6	0.9	1.4	3.4	3.5	3.2
	Δ*δ*(VK)	0.7	0.3	0.3	1.6	3.4	3.4	3.5
	*χ*(F)	0.4	0.4	0.5	0.7	2.3	2.4	2.1
	*χ*(VK)	0.4	0.4	0.4	0.8	2.4	2.4	2.5
	EUC-d	6.0	7.3	1.9	1.8	3.6	4.0	4.1

**Table 5:** Polymer ranks order provided by predictive and spray drying methods for the production of IBU, NAP, CAR, and ITR ASDs. Evaluation of prediction accuracy with the probability calculation of each method to predict the polymer nature (*f*1) and the polymer rank order (*f*2) of spray-dried amorphous solid dispersions (SDASDs).

Predictive and Manufacturing Methods		IBU	NAP	CAR	ITR
***Δδ*(F)/*χ*(F)**	Polymer ranking	EUD > SOL > EUD55	EUD55 > SOL > EUD	PK30 > HP50 > HAS	HP50 > HAS > K30
	*f*1/*f*2	0/0.33	0.33/0.33	0/0.33	0/0.33
***Δδ*(VK)/*χ*(VK)**	Polymer ranking	EUD55 > EUD > SOL	EUD55 > EUD > SOL	PVA > HAS > PK30	HAS > K30 > HP
	*f*1/*f*2	0.33/0.33	0/0.33	0/0.33	0.33/0.33
**EUC-d**	Polymer ranking	SOL > EUD55 > EUD	SOL > PVA > EUD	PVA > PK30 > SOL	PVA > PK30 > SOL
	*f*1/*f*2	0/0.33	0.66/0.66	0.33/0.33	0/0
**Film casting-RT**	Polymer ranking	PK30 > PVA > SOL	SOL > PK30 > HAS	EUD > PK30 > HAS	EUD > EUD55 > HP50
	*f*1/*f*2	1/1	0/0.33	0.66/0.66	1/1
**Film casting-reduced pressure**	Polymer ranking	PVA > PK30 > SOL	SOL > PVA > PK30	PK30 > EUD > HAS	HAS > HP50 > EUD55
	*f*1/*f*2	0.33/1	0.33/0.66	0/0.66	0/0.66
**Quench cooling**	Polymer ranking	PK30 > PVA > HAS	PK30 > PVA > SOL	PK30 > PVA > SOL	EUD > EUD55 > HAS
	*f*1/*f*2	0.66/0.66	0.66/0.66	0/0.33	0.66/0.66
**Atomization device**	Polymer ranking	PK30 > PVA > SOL	PK30 > PVA > SOL	EUD > PK30 > EU55	EUD > EUD55 > HP50
	*f*1/*f*2	1/1	0.66/0.66	1/1	1/1
**Spray dryer**	**Polymer ranking**	**PK30 > PVA > SOL**	**PK30 > PVA > EUD**	**EUD > PK30 > EUD55**	**EUD > EUD55 > HP50**
